# Systematic Review of the Interaction between Nutrition and Immunity in Livestock: Effect of Dietary Supplementation with Synthetic Amino Acids

**DOI:** 10.3390/ani11102813

**Published:** 2021-09-27

**Authors:** Laura Montout, Nausicaa Poullet, Jean-Christophe Bambou

**Affiliations:** INRAE UR143, Unité de Recherches Zootechniques, Centre INRAE Antilles Guyane, Domaine de Duclos, Prise d’Eau, 97170 Petit Bourg, Guadeloupe, France; laura.montout@inrae.fr (L.M.); Nausicaa.poullet@inrae.fr (N.P.)

**Keywords:** nutrition, immunity, synthetic amino acids

## Abstract

**Simple Summary:**

Nutritional manipulation of livestock diets has long been considered asa tool for the control of infectious diseases. The objective of this literature review is to provide a comprehensive and critical view of the studies that have investigated the interactions between synthetic amino acids supplementation and immune response against infectious diseases in livestock. We performed a literature search in two main databases, PubMed and Web of Science. Based on our criteria for eligibility of the research articles, we selected 58 studies. Most of the studies selected focus on poultry and three amino acids that are all associated with a significant improvement in host response: methionine, threonine and arginine. The most described immune mechanisms associated with synthetic amino acids supplementation were directed against intracellular pathogens. We highlight the need for more analytical studies using these three amino acids, particularly in livestock other than poultry, and their use with other types of pathogens.

**Abstract:**

Infectious diseases represent one of the most critical threats to animal production worldwide. Due to the rise of pathogen resistance and consumer concern about chemical-free and environmentally friendly productions, the use of antimicrobials drugs is no longer desirable. The close relationship between nutrition and infection has led to numerous studies about livestock. The impact of feeding strategies, including synthetic amino acid supplementation, on host response to various infections has been investigated in different livestock animals. This systematic review provides a synthesis of the experimental studies on the interactions between synthetic amino acid supplementation and immune response to infectious diseases in livestock. Following PRISMA guidelines, quantitative research was conducted using two literature databases, PubMed and Web of Science. The eligibility criteria for the research articles were: (1) the host is a livestock animal; (2) the supplementation with at least one synthetic amino acid; (3) at least one mediator of immunity is measured; (4) at least one production trait is measured. Data were extracted from 58 selected studies. Articles on poultry were the most numerous; few contained experiments using ruminants and pigs. Most of the authors hypothesized that synthetic amino acid supplementation would particularly improve the animals’ immune response against intracellular pathogens. An increase in T and natural killer lymphocytes and macrophages activation, intracellular redox state, lymphocytes proliferation and antibodies production were the most described immune mechanisms associated with synthetic amino acid supplementation. Most of the selected studies focused on three amino acids (methionine, threonine and arginine), all of which are associated with a significant improvement of the host immune response. The use of synthetic amino acid supplementation appears as an encouraging perspective for livestock infectious disease management, and research must concentrate on more analytical studies using these three amino acids.

## 1. Introduction

Infectious diseases are one of the most significant threats to livestock farming worldwide. These diseases have important economic impacts and can lead to food insecurity by increasing morbidity and mortality, reducing market value and productivity [1,2]. The primary strategy for reducing the spread of pathogens on farms relies on the use of chemical drugs (e.g., antibiotics and anthelmintics). However, this strategy involving pharmaceutical treatments of livestock as the sole method of infectious disease control is no longer desirable for sustainable production due to the rise in drug-resistant pathogens worldwide, together with concerns about the presence of drug residues in edible animal products and as contaminants in the environment [3,4]. Moreover, antimicrobial-resistant pathogens represent a major concern impacting public health, particularly with the transfer of multi-resistant bacteria from animals to humans [5]. This issue also raises questions about animal welfare, given the close relationship between animal health and welfare. Consequently, the control of infectious diseases in livestock has to be included in a global scheme of management in which alternative control strategies have to be developed complementary to a parsimonious use of classical practices in accordance with agroecological concepts [6,7]. Indeed, in the context of global change, the aim is to sustain the economic and environmental viability of agricultural systems closely linked to their local ecosystem [8].

A vaccination strategy to improve livestock immunization against specific pathogens is a vital tool for the efficient prevention of infectious diseases. However, vaccination is not available for all infectious diseases of importance. Some vaccines have a number of shortcomings with regard to safety, efficacy and/or user-friendliness that limit their effectiveness [9]. The genetic selection to reduce host susceptibility is a promising strategy, but it is feasible only in the long term and requires operational extension services and is consequently costly [10,11]. Therefore, short-term control strategies, such as tailored management of animal nutrition, are also necessary.

In recent decades, knowledge regarding livestock nutrition has considerably evolved in terms of improvements in feed efficiency, whatever the production considered. Most of the time, these nutritional strategies to maximize production performances were developed in healthy, un-challenged animals. However, it has long been shown that optimal immune function is dependent on an adequate supply of protein, energy and micronutrients (e.g., vitamins and minerals), thus highlighting the close relationship between host nutrition and the immune system [12].

In animal production, the key role of dietary protein and amino acids, which are the building blocks of proteins, on different functions of the immune system, has been investigated [13,14,15]. Indeed, numerous studies were conducted to find an optimal requirement of amino acids by different livestock animals, including birds [16], pigs [17] and ruminants [18], under numerous developmental, environmental, pathological and nutritional conditions. Amino acids play an important role in regulating immune responses, including the activation of lymphocytes, NK cells and macrophages; proliferation of lymphocytes; regulation of intracellular redox balance; gene expression; and production of cytokines [19]. Thus, the availability of specific dietary amino acids is essential for the control of infectious diseases, including viral infections. This review aimed to provide insight into the current knowledge regarding the effect of dietary supplementation with synthetic amino acids on the immune response to infectious diseases in livestock animals.

## 2. Materials and Methods

**Figure 1:**
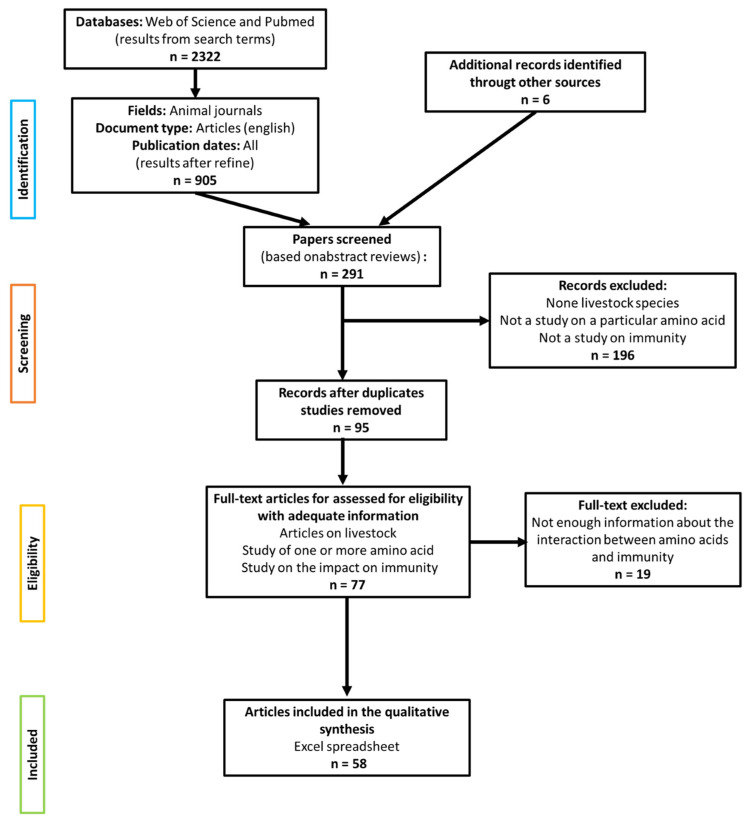
Search strategies: PRISMA Flow Diagram (2009). Flowchart describing the methods of data collection and article selection.

The study methodology was based on the guideline of “Preferred Reporting Items for Systematic Reviews and Meta-Analyses: The PRISMA Statement” [20]. The literature search was conducted using the Web of Science and PubMed electronic databases. The search strategy is described in Figure 1 and the search terms used are in Table 1.

**Figure 2:**
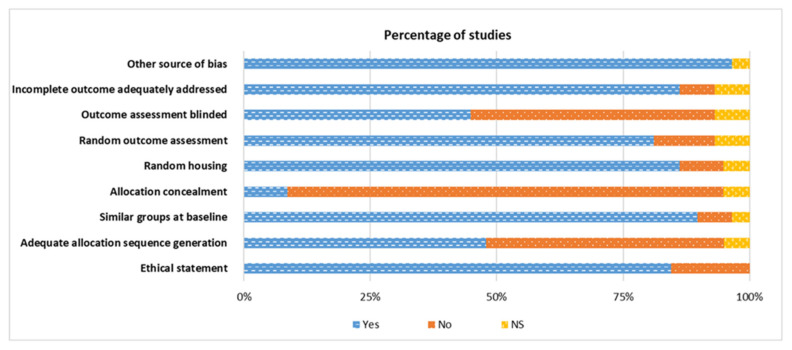
Qualitative evaluation of the risk of bias for the studies included in the systematic review. Yes: Percentage of studies scoring with low risk of bias. No: Percentage of studies scoring with high risk of bias. NS: Percentage of studies that did not specify the key methodological variables.

*The use of truncation (*) allows a query to be made only on the root of the word. Furthermore, the reference lists from selected articles provide additional articles. Studies were closely evaluated and selected for inclusion if they were performed using livestock to measure the effect of dietary supplementation (above the National Research Council, NRC requirement). They had to have at least one particular synthetic amino acid on the expression of at least one mediator of the immune response following an immune challenge, and measure at least one production trait. Information extracted from each study included livestock types, immune challenge (live pathogens, microbe-associated-molecular-pattern, non-pathogenic antigens, non-specific immune stimulators), immune mediators and the impact on their expression (e.g., humoral and cellular mediators, lymphoid organ weight), dietary synthetic amino acid supplementation (quantity), basal diets, number of animals per group and production traits (Table 2). A total of 30 studies out of 58 showed a significant effect on the expression of at least one immune effector. The methodological quality of the included studies was evaluated qualitatively with a 9-point scoring system based on SYRCLE’s risk of bias tool (Figure 2) [21].

## 3. Results

### 3.1. Study Selection and Characteristics

A total of 2322 results were provided by the Web of Science and PubMed databases (Figure 1 and Table 1). After excluding duplicates and screening titles and abstracts, 77 full-text research articles were further evaluated for relevance. Finally, 58 research articles were included in the qualitative synthesis. All included research articles were in English and published between 1969 and 2020. Six papers were not found using the bibliographical research strategy described but were located in the citations of the selected articles.

### 3.2. Synthesized Findings

**Figure 3:**
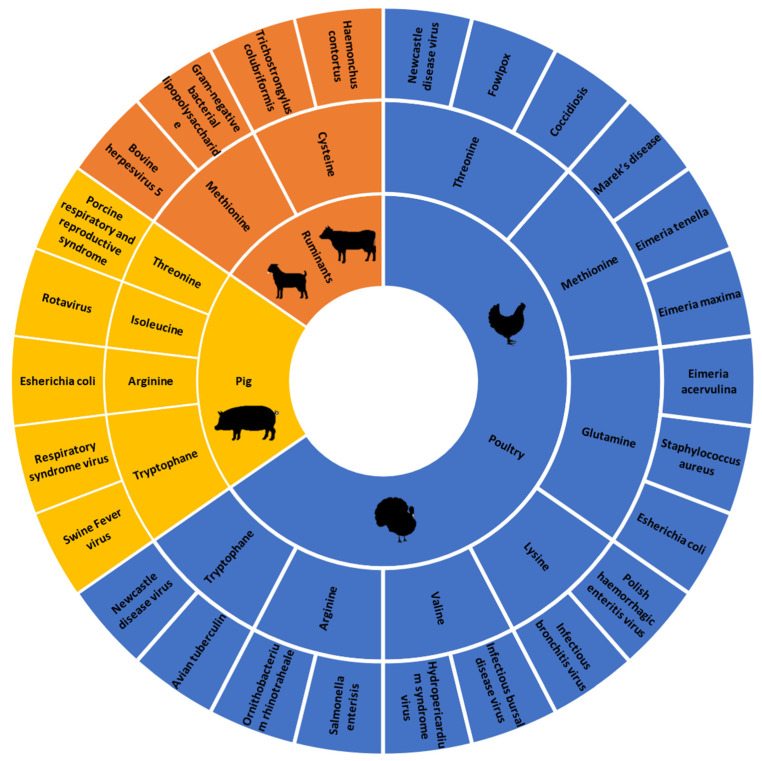
Repartition of livestock, amino acids and immune stressors among the selected articles.

The main objective of this systematic review was to synthesize the knowledge on the effect of dietary supplementation with synthetic amino acids on the immune response to infectious diseases in livestock. The bibliographical research was focused on all livestock, but only three types were found: poultry, ruminant and pig. The research articles on poultry were the most numerous compared to pigs and ruminants (48 out of 58 articles), and chicken was the main poultry studied. One of the major inclusion criteria was monitoring an immune challenge, including live bacteria, virus, parasite and their associated molecular pattern. The studies using non-specific immune stimulators (e.g., phytohemagglutinin, concanavalin A) were excluded. Among the 20 proteinogenic amino acids, only 9 were found in the selected research articles. The most studied amino acids were methionine (24 articles), followed by arginine (19 articles) and threonine (12). Figure 3 represents the amino acids used as supplementation, the type of livestock and the pathogens with their associated molecular pattern used in the 58 studies.

### 3.3. Assessment of Risk of Bias

The results of the qualitative evaluation of the risk of bias are presented in Figure 2. An ethical statement about animal treatment in the research was not included in 25% of the studies. Most of these research articles were published before this statement was mandatory. An adequate allocation sequence generation was stated in 50% of the studies, while 24% did not use random housing and 94% reported using similar groups at baseline. However, allocation concealment was not performed in 87% of the studies. Random outcome assessment was stated in 80% of the studies, but the outcome assessment was not blind in 57% of the studies. An incomplete outcome was adequately addressed in 92% of the studies, and other sources of bias were described in 98% of the studies.

## 4. Discussion

The fine-tuning of nutritional strategies to support optimal function of the immune system in livestock is of great interest for the development of sustainable management in animal health. The immune response against invading pathogens is expensive in terms of proteins and calories because of the energy and protein requirements of immune cells for the synthesis of immune mediators and repairing damaged tissue [52]. Moreover, several vitamins (e.g., A, B6, B12, C, D, E and folate) and trace elements (including zinc, iron, selenium, magnesium and copper) play important and complementary roles in the development of both the innate and the adaptive immune systems. A short-term and easy-to-implement strategy consists of improving immune functions through animal nutrition. In the available literature, dietary supplementation with synthetic amino acids appears as a pertinent lever for preserving and regulating the immune response against pathogens.

### 4.1. Role of Amino Acids in Immune Response

#### 4.1.1. Sulphur-Containing Amino Acid (SAA)

Methionine and cysteine are the two principal sulphur-containing amino acids used as a substrate for proteins biosynthesis [53]. These two amino acids are also precursors of numerous metabolites implicated in various physiological functions including the immune system [16,54,55]. Methionine is a methyl group donor participating in both transmethylation and remethylation pathways, including the methylation of DNA and proteins and the regulation of gene expression [22]. The implication of methionine in the cellular immune response and the humoral immune response has been suggested in numerous studies [56].

In turkeys and broilers, it has been shown that higher dietary methionine levels (from 0.60% and 0.90% in turkeys and broilers, respectively) increased the level of methionine in the peripheral blood, and was correlated with higher levels of peripheral leucocytes and IgG after vaccine challenges [22,31,32]. In addition, increased levels of CD4+ and CD8+ T cell subpopulations and IgM+ B subpopulation in immune organs, such as the thymus and the bursa of Fabricius, were observed in turkeys [31,32]. Higher antibody levels were also observed in broilers supplemented with a combination of 0.65% methionine and 0.13% of choline [24]. Indeed, methionine is a substrate for choline, and thus acetylcholine and phosphatidylcholine synthesis play a central role in numerous metabolic pathways including leucocyte metabolism. In contrast, despite the use of rumen-protected dietary methionine, no effect on the immune response after a vaccine challenge against the herpesvirus was observed in beef heifers [57]. Cysteine is involved in the formation of interchain and intrachain disulphide bonds of proteins. In vitro studies showed that cysteine and other cysteine derivatives can modulate lymphocyte and macrophage functions [58]. In broiler chickens supplemented with 0.65% of cysteine, an increase in immune and the inflammatory responses was shown after an *Escherichia coli* LPS (lipopolysaccharide) injection, notably by the production of several cytokines (IL-1, IL-6 and TNF-α) by macrophages [28]. The supplementation with a combination of methionine and cysteine (from 0.8%) was also associated with an increase in the level of anti-*Eimeria* IgA in broilers [30].

Few studies have investigated the role of cysteine in the improvement in immune responses. The bibliographic analysis performed here showed only one article in which the impact of cysteine supplementation on the immune response of sheep against *Haemonchus contortus* and *Trichostrongylus colubriformis* was studied. Cysteine was reported to increase blood eosinophil counts and globule leucocytes in the abomasal mucosa following experimental infection [47]. Further studies need to be conducted in other types of livestock to complete investigations on metabolic cysteine pathways in response to infectious diseases. Despite the great advances in our knowledge of sulfur-containing amino acids, there are important areas where further work is required. Cysteine appears to influence certain aspects of immunocompetency in sheep, although the exact role of cysteine in the relationship between wool production and parasite susceptibility requires further elucidation.

#### 4.1.2. Amino Acid Amide

The interaction between dietary glutamine and immunity has been well studied in mice [59]. Glutamine is implicated in purine and pyrimidine biosynthesis in tissues of the immune system [60], in lymphocyte proliferation [61] and in cytokine production [62]. In dairy livestock, glutamine is usually considered a good feed additive to improve the amino acids profile of milk due to the presence of casein in glutamine residues [19]. Dietary supplementation of broiler chickens with glutamine (3%), arginine (2%) and threonine (2%) was associated notably with an increased number of goblet cells, a lower level of mucosal IgA and a reduction in the thymus, suggesting an improved immune response against a co-infection with *Eimeria* and *E. coli* [41]. Similar to the results of Bartell and Bartal (2007) [63], this higher level of dietary glutamine in broiler was associated with lower levels of mucosal IgA, probably due to the rapid and efficient immune response against the parasitic infection.

#### 4.1.3. Basic Amino Acid

The basic amino acids, lysine and arginine, play important roles in membrane protein activity and the actions of antimicrobial, toxin and cell-penetrating peptides. In poultry, due to the absence of a functional urea cycle, arginine is an essential amino acid [64]. Many studies demonstrated the importance of arginine supplementation above the NRC recommendations to support growth performance and improve the host immune response against avian infectious diseases [23,39]. In these studies, poultry challenged with viruses such as infectious bronchitis virus (IBV), Newcastle disease virus (NDV) or infectious bursal disease (IBD) were able to establish an immune response with no impact on the growth rate.

Arginine metabolism has been described as a key regulator of innate and adaptive immunity. Cells of the myeloid lineage, such as macrophages and dendritic cells, could modulate the immune response by regulating the expression of two enzymes: NOS (nitric oxide synthase) and arginase [65]. NOS uses arginine as a substrate to produce NO (nitric oxide), a key molecule involved both directly and indirectly in host immune response [66]. In broiler chickens, it has been shown that dietary arginine (from 0.45% above the NRC recommendation) may modulate macrophage phagocytosis by induction of the expression of key cytokines, particularly IL-1, IL-2, INF-γ and TNFα [36,38]. In piglets challenged with *Escherichia coli* LPS (lipopolysaccharide), arginine supplementation (0.5% above the NRC recommendation) increased the infiltration of ileal mucosa by IgA-secreting cells, CD8+ and CD4+ T cells [43]. Many pieces of evidence demonstrated that a dietary deficiency in lysine decreased the synthesis rate of proteins (including cytokines) and lymphocytes proliferation. The impact of dietary supplementation with lysine on the immune response is always studied in association with another amino acid. In broiler chickens, dietary supplementation with lysine and methionine improved antibody production against Newcastle and the Gumboro diseases [49]. This positive effect on the immune response, together with performance production, was also observed in piglets challenged with *Escherichia coli* K88 [50]. An average optimal standardized ileal digestible tryptophan: lysine ratio of 21% optimized the performance of the challenged piglets and increased the expression of IL-10 in the ileum.

#### 4.1.4. Branched-Chain Amino Acid (BCAA)

Branched-chain amino acids (BCAA), which include leucine, isoleucine and valine, contribute notably to the synthesis of glutamine in the skeletal muscle and are essential for lymphocytes’ proliferation in response to immune stimulation [67]. Thus, dietary BCAA restriction impairs several aspects of the immune function and increases the susceptibility to different pathogens [68]. In broiler chickens, it has been shown that valine, which constitutes approximately 18% of muscle myofibrillar protein, could be associated with a decrease in the weight of lymphoid organs [69,70]. Furthermore, studies have demonstrated that birds subjected to either killed or live viruses and supplemented with a high level of dietary valine showed increased antibody production. In weaned piglets, isoleucine and valine (0.19 and 0.27% respectively) supplementation to protein-restricted diets protected villous morphology and increased levels of intestinal immunoglobulins [71]. Piglets are particularly sensitive to rotavirus infection that leads to severe gastroenteritis [72]. Mao et al. [51] showed that adding isoleucine in pig diet increases the production of IgA, IgG, several cytokines (IL-1β, IFNβ, IFNγ, TNF-α and IL-10) and β-defensins in serum, ileum and/or mesenteric lymph [51].

#### 4.1.5. Other Essential Amino Acids (OAA)

Threonine is implicated in many biological functions, such as, body protein synthesis, collagen, antibody and uric production or pancreatic enzymes [73]. In broilers, threonine is considered an essential amino acid because of its impact on growth performance, gut health, carcass traits and immune functions [74]. Dietary threonine supplementation, above the requirements, promotes the growth of immune organs and stimulates the synthesis of immunoglobulins and antibodies in broiler chickens under immune stress [45,46,75]. Indeed, it has been suggested that, in broiler chickens, threonine would be especially implicated in the regulation of the immune system response by controlling the gut microbial population and the production of IgA and IgG [44]. In pigs, during the post-weaning period, due to the high susceptibly to intestinal bacterial infection, a higher requirement of threonine is recommended. Interestingly, threonine is the most abundant amino acid in mucin proteins produced by the intestinal mucosa, [76]. In addition, in a recent study, a high level of dietary threonine (0.9%) increased the level of IgA production against an enterotoxigenic *E. coli* K88 (ETEC) [77]. In broiler chickens, following avian tuberculin injection, dietary supplementation with tryptophan (0.3 and 0.5%) improved total oxidant status and overall antibody response, together with cellular immunity, [48]. The antibody response and the IgG increased when the tryptophan level exceeded the NRC requirements. The combination of tryptophan and arginine from two times the NRC levels in starter, grower and finisher diets of broiler chickens has an efficient immunomodulatory impact on virus infections [33]. Indeed, this treatment improved both the innate (IFNα) and humoral (IgG) immune responses against the infectious bursal disease virus (IBDV).

**Figure 4:**
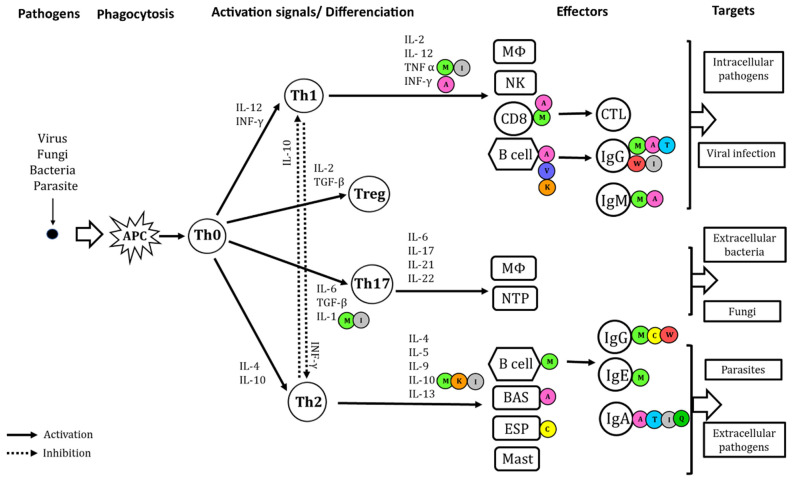
Amino acids suggested role in the host (mainly poultry) immune response against pathogens. Abbreviation: intracellular and extracellular antigens; ACP, antigen-presenting cells, generally dendritic cells; Th0, naive T helper cell carrying CD4 marker; Th1, Th2, helper T cells; Treg, regulatory T cell; IL, interleukin; IFNγ, interferon γ; IFNα, interferon α; TGF-β, Transforming growth factor β; TNFα, tumor necrosis factor α; MΦ, macrophage; CD8, CD8 T cell carrying CD8 maker; CTL, cytotoxic T cells; B cell, B lymphocytes; NK cell, natural killer cell; NTP, neutrophil; ESP, eosinophil; BAS, basophil; Ig, immunoglobulin; Mast, mastocyte. Impacts of amino acids.

Most of the selected studies focused on three amino acids: methionine, threonine and arginine, which were all associated with a positive impact on the host immune response. The mechanisms of immunity improved by dietary amino acids in livestock animals involved both Th1 and Th2 responses, and Th17 to a lesser extent. We have summarized the effects of the different amino acids studied in the selected research articles in a schematic representation of the immune response (Figure 4). 

The most important effects of amino acids on immunity were mostly observed at a high level of supplementation. The majority of the studies were on chicken, and few studies investigated the effect of amino acid supplementation on ruminant immune response. This may be due to the fact that ruminant physiology limits amino acid availability. To overcome this limitation, the utilization of rumen-protected amino acids is of interest. Therefore, there is a need for more studies in pigs and ruminants with live pathogens to increase our knowledge on the effect of dietary supplementation with synthetic amino acids on the immune response against infection.

## Figures and Tables

**Table 1:** Search terms used for the systematic review.

Categories	Search Terms
Population	(livestock OR ovine OR ruminant OR cattle) AND (“small ruminant” OR goat OR ewe OR sheep OR lamb) AND (calf OR beef OR cow) AND (porcine OR pig OR piglet OR sow) AND (poultry OR chicken OR chick OR hen OR turkey OR broiler) AND (rabbit OR duck)
Nutrition	(“amino acid” OR protein) AND (supplement * OR treatment) AND (nutrition OR nutrient) AND (methionine OR cysteine OR each of the 20 proteinogenic amino acids)
Immunity	(immune * OR “immune system” OR (‘‘immune function’’ OR “humoral immunity” OR “cellular immunity” OR “immunological response”) AND (infection OR infected OR “infectious disease”) AND (parasite * OR nematode *) AND (resistance OR resistant)

**Table 2:** Main amino acids impact on immune effectors in livestock during an infectious stress. Only studies showing a significant increased effector expression are listed.

Amino Acids	Livestock	Pathogens	Immune Effectors Monitored	References
Methionine	Poultry	Virus	Antibody titer Immunoglobulin G Lymphocyte and heterophil	[22]
			Total antibody	[23]
			Antibody titer	[24]
			Antibody titer Immunoglobulin G	[25]
			Antibody production	[26]
		Bacteria	Antibody titer	[27]
			Cytokine IL-1	[28]
			Cytokine TNFα and IL-1	[29]
		Parasite	Cytokine IFNγ and IL-10 Immunoglobulin G and A	[30]
	Turkey	Bacteria	Immunoglobulin M CD8+ T cells	[31]
			Immunoglobuline G	[32]
Arginine	Chicken	Virus	Cytokines IFN-α/γ Immunoglobulin G	[33]
			Antibody titer	[23]
			B cells	[34]
			Lymphocyte proliferation Basophil hypersensitivity	[35]
			Cytokines IFN-α/γ Immunoglobulin G	[36]
			Lymphocyte and antibody	[37]
			Immunoglobulin A and M Cytokine INF-α Lymphocytes proliferation	[38]
			T cells and B cells Lymphocytes CD4+ and CD8+	[39]
		Bacteria	Immunoglobulin M	[40]
			Immunoglobulin A regulation	[41]
		Parasite	Immunoglobulin A production	[42]
	Pig	Bacteria	Immunoglobulin A CD8+ and CD4+ T cells production	[43]
Threonine	Poultry	Virus	Antibody production Immunoglobulin A and G secretion	[44]
			Antibody responses	[45]
			Antibody production	[46]
		Bacteria	Immunoglobulin A regulation	[41]
Cysteine	Sheep	Parasite	Eosinophil Immunoglobulin G	[47]
Tryptophan	Chicken	Bacteria	Antibody titer Immunoglobulin G	[48]
		Virus	Cytokine INF-α Immunoglobulin G	[33]
Valine	Chicken	Virus	Antibody production	[26]
Lysine	Chicken	Virus	Antibody and lymphocytes	[49]
	Pig	Bacteria	Cytokine IL-10	[50]
Isoleucine	Pig	Virus	Immunoglobulin A and G Cytokine IL-1β, IFN-β, IFN-γ, TNF-α and IL-10	[51]
Glutamine	Chicken	Parasite	Immunoglobulin A	[41]

## Data Availability

No new data were created or analyzed in this study.
